# PRCnet: An efficient model for automatic detection of brain tumor in MRI images

**DOI:** 10.1371/journal.pone.0292768

**Published:** 2025-12-15

**Authors:** Ahmeed Suliman Farhan, Muhammad Khalid, Umar Manzoor

**Affiliations:** 1 Electronic Computer Center, University of Anbar, Ramadi, Iraq; 2 School of Computer Sciences, University of Hull, Kingston upon Hull, United Kingdom; 3 School of Engineering, Computing and Mathematical Sciences, University of Wolverhampton, Wolverhampton, United Kingdom; IPM: Institute for Research in Fundamental Sciences, IRAN, ISLAMIC REPUBLIC OF

## Abstract

Brain tumors are the most prevalent and life-threatening cancer; an early and accurate diagnosis of brain tumors increases the chances of patient survival and treatment planning. However, manual tumor detection is a complex, cumbersome and time-consuming task and is prone to errors, which relies on the radiologist’s experience. As a result, the development of an accurate and automatic tumor detection system is critical. In this paper, we proposed a new model called Parallel Residual Convolutional Network (PRCnet) model to classify brain tumors from Magnetic Resonance Imaging. The PCRnet model uses several techniques (such as filters of different sizes with parallel layers, connections between layers, batch normalization layer, and ReLU) and dropout layer to overcome the over-fitting problem, for achieving accurate and automatic classification of brain tumors. Our methodology used data augmentation techniques such as rotation, flipping, and scaling. These enhanced the diversity and quantity of the training dataset, contributing significantly to the model’s improved performance. The PRCnet model is trained and tested on two different datasets and obtained an accuracy of 94.77% and 97.1% for dataset A and dataset B, respectively which is way better as compared to the state-of-the-art models. Our PRCnet code publicly available at: https://github.com/Ahmeed-Suliman-Farhan/PRCnet-Model

## Introduction

The brain is a complicated and essential organ of the human body [1] and is responsible for controlling the entire nervous system [2]. Due to its intricate design, even a slight malfunction in the brain cells can cause severe disruption in the performance of connected organs [3]. A brain tumor is a disease that occurs due to the abnormal growth of brain cells [4]. This uncontrolled growth of cells poses severe problems to human health [5]. Brain tumors also come in different forms, and their symptoms are likely to vary depending on the type of tumor [6]. Even though brain tumors comprise only 2% of all cancers, they are disproportionately to blame for cancer-related fatalities [7].

**Fig 1:**
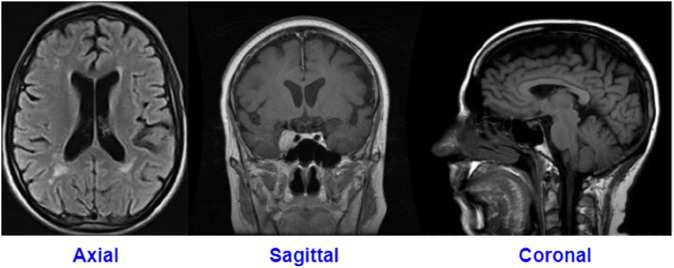
MRI image planes (this MRI sample from dataset A and dataset B).

The tumor location and growth rate can affect the nervous system functionality differently and may cause headaches, seizures, memory loss, balance problems, etc [3]. There are two types of brain tumors namely malignant and benign tumors [8]. Malignant tumors are considered to be the most perilous. They tend to grow rapidly, infiltrate surrounding tissue aggressively, spread to other areas in the brain, and can cause severe damage to the nervous system [9]. On the other hand, benign tumors tend to grow at a slower pace [10]. MRI is widely used as a non-invasive diagnostic tool for detecting various types of brain tumors and provides detailed information on the brain tissues [11]. Also, It is one of the best and most accurate tests for tumor identification due to its ability to produce high-quality images of human anatomy [12]. MRI uses radio waves and magnetic resonance to retrieve detailed brain information and can help doctors in tumor diagnosis [13]. The brain can be imaged in three planes, coronal, axial and sagittal - see Fig 1 and multi-angle images can be obtained [14].

Early detection of tumors is crucial in identifying the severity of the brain tumor, which is essential for deciding the most appropriate treatment plan [15]. In addition, accurately identifying the severity of a brain tumor can assist medical professionals in making informed decisions related to treatment options [16]. Also, it increases the chance of patient survival and improved quality of treatment [17]. The task of manually detecting tumors is arduous, intricate, and time-consuming, and may often be erroneous owing to the diverse nature of cancer cells. Additionally, it relies on the radiologist’s expertise [18]. As a result, it is imperative to develop a dependable automated tumor detection and segmentation technique that can effectively assist radiologists with prompt and efficient classification [19].

Medical image processing is critical in assisting healthcare professionals in diagnosing a wide range of medical conditions [20]. In conjunction with magnetic resonance imaging (MRI), biomedical image processing facilitates the detection and segmentation of brain tumors [21]. Deep neural networks have emerged as a powerful tool for processing medical images, achieving remarkable results in various applications [22]. In addition, the use of deep neural networks for detecting brain tumors from MRI scan images has become increasingly popular due to their high accuracy [23]. Convolutional Neural Network (CNN) is one of the most effective deep learning designs for learning representations from an input signal with different levels of abstraction [24]. CNN is a technology with many advantages over traditional machine learning algorithms. The benefits include a) ability to perform complex feature extraction directly from raw data, significantly reducing the need for manual feature engineering [25], and b) infer hidden patterns from complex data and images [26]. Using an intelligent, fast and error-free system for detecting brain tumors using MRI images is very important to help clinicians accurately detect the tumors [27].

The CNN has performed exceptionally well in image classification tasks and has achieved better performance than rival human experts in classifying medical images [24]. For example, Aurna et al. proposed a new approach by using a two-stage feature ensemble of CNN to classify the brain tumors. By experimenting and testing the models (VGG19, EfficientNetB0, InceptionV3, ResNet50, Xception, and Scratched CNN) on three datasets of MRI images, the models EfficientNetB0, ResNet50, and Scratched CNN are selected for the first stage of the ensemble. The two models with the highest accuracy are selected for the second stage of the ensemble. The proposed model are trained and tested on three databases and the fourth dataset resulted from merging the three datasets, and achieved average accuracy of 99.13%[28]. Although the accuracy obtained is very good, however, the proposed model when tested on dataset A and B, obtained 92.59% and 94.2% accuracy respectively. This difference in results is due to difference in 1) datasets and 2) datasets splitting into groups for testing, validation, and training. Furthermore, the model is considered very complex because it combines three models, two of which are designed to classify a thousand classes from the Imagenet dataset [29].

In this paper, we proposed the state of the art model called PRCnet, to overcome the limitations and problems that exist in the literature. The existing models are either very simple (obtain good accuracy on very small dataset, but under-perform on large dataset) or very complex (incorporate a number of models that are essentially not designed to handle medical images). All this motivated us to develop a new model that uses parallel layers with different filter sizes and connections between layers to provide excellent accuracy in classifying brain tumors. The main contributions of the PRCnet model are:

- A novel model called PRCnet for the detection of brain tumors in MRI images.
- Computationally less expensive where total parameters for PRCnet model are 21413060 as compared to 144853060, 35533911, 134276932 for Musallam et al. [30], Aurna et al. [28], and VGG16 [31] respectively.
- Different filter sizes used with parallel layers help the model to recognise small and large features which leads to improved accuracy.
- A comprehensive comparison is performed with state-of-the-art models, the PRCnet model achieved an accuracy of 97.1 and 94.77 for datasets B and A respectively.
- The PRCnet robustness is validated by using a cross-validation technique on datasets A and B.

This paper is organized into several sections following this introduction. The Literature Review section provides a detailed overview of prior studies, categorizing studies into ‘Novel Models from Scratch’ and ‘Based on Standard Transfer Learning Models’, and discusses the limitations of existing studies. The Proposed Methodology section explains the design and development of the proposed PRCnet model. The Experimental Results section presents the performance evaluation and comparative analysis. The Discussion section interprets the findings and their implications. Finally, the Conclusion section summarizes the main contributions of this study and outlines possible directions for future work.

## Literature review

The application of deep learning techniques in medical imaging, particularly MRI brain tumor diagnosis, has seen considerable advancements in recent years. Researchers have developed various models that enhance diagnostic accuracy and processing speed, enabling doctors to make timely clinical decisions. This review categorises recent studies into novel models created from scratch and enhancements made to existing models with transfer learning.

### Novel models from scratch

This section highlights studies introducing new deep-learning models explicitly tailored for MRI brain tumour detection. For example, Ullah et al. [32] It developed TumorDetNet, a novel model to enhance tumor detection in medical imaging. The research aims to enhance the precision and effectiveness of tumour identification by incorporating sophisticated deep-learning methods. The methodology utilises a specialised neural network architecture incorporating many convolutional and dense layers. This architecture is specifically designed to enhance the extraction of features and improve the accuracy of classification tasks. The results demonstrate that TumorDetNet surpasses existing models in terms of detection accuracy. TumorDetNet was trained and assessed using six established Kaggle brain tumour MRI datasets to detect and categorise brain tumours as either malignant or benign. It also distinguishes between glioma, pituitary, and meningioma tumours. The TumorDetNet demonstrated a detection accuracy of 99.83% in identifying brain tumours. It reached a perfect classification accuracy of 100% in distinguishing between malignant and benign tumours. It also achieved a classification accuracy of 99.27% in discriminating among pituitary, glioma, and meningioma tumours.

Aurna et al. [28] proposed a new approach by using two-stage feature ensemble of CNN to classify the brain tumors. By experiment and testing the models (VGG19, EfficientNetB0, InceptionV3, ResNet50, Xception, and Scratched CNN) on three datasets of MRI images, the models EfficientNetB0, ResNet50, and Scratched CNN are selected for the first stage of the ensemble. The two models with the highest accuracy are selected for the second stage of the ensemble. The proposed model is trained and tested on three databases and the fourth dataset resulted from merging the three datasets, and achieved average accuracy of 99.13%.

Rai and Chatterjee [33] proposed the CNN model (LeUNet), which is less complex, has fewer layers, and faster processing time for detecting brain tumors. The proposed model is simulated on a dataset containing 253 images and compared with VGG-16, U-Net, and Le-Net, where the accuracy of proposed model is 98% and 94% on cropped and uncropped images respectively, and a speedy processing time of only 252.36 seconds. However, the model is not tested on a larger database as well as it did not classify the types of brain tumors.

Abd El Kader et al. [34] proposed a differential deep convolutional neural network model that uses magnetic resonance images to classify different types of brain tumors. The authors used CNN architecture’s differential operators to create extra differential feature maps in the original CNN feature maps to improve the proposed model. This enables model to analyze the pixel orientation pattern based on contrast calculations and classify large number of images with high accuracy. A dataset of 25,000 MRI images collected from Tianjin Universal Center of Medical Imaging and Diagnostic (TUCMD) is used for training and testing. The model achieved 99.25% accuracy, 95.23% F-score, 95.89% sensitivity, 97.22% precision, and 93.75% specificity. However, the model is not evaluated on a dataset that contains more than two classes (tumor types).

Irmak [35] proposed three CNN models for three different classification tasks, the first one classify tumor presence, the second one classify tumor into five types (metastatic, normal, meningioma, glioma and pituitary), and finally, the third one classify the tumor in three grades (Grade II, Grade III and Grade IV). The proposed models achieved 99.33%, 92.66% and 98.14% accuracy respectively. The accuracy is high for tumor detection, however, accuracy decrease significantly for tumor classification.

Díaz-Pernas et al. [36] proposed a model based on multiscale convolutional neural network for automatic tumor segmentation and classification. The input images are processed at three spatial scales along with different processing paths. The proposed model is trained and tested on a database containing 3064 MRI images, including three types of tumors (glioma, meningioma and pituitary tumor).The method achieved an accuracy of 97.3% in classifying brain tumors, also obtained distinct segmentation performance, an average of 0.940, 0.828 and 0.967 for sensitivity, Dice index and pttas value respectively. However, the model lacks durability analysis and testing on a larger dataset.

Musallam et al. [30] proposed three preprocessing steps to improve the MRI brain images, such as removing the confusing variables, denoising the MRI images, and enhancing the contrast of these images. In addition, a new Deep Convolutional Neural Network (DCNN) architecture is proposed to effectively diagnose three types of brain tumor (glioma, meningioma, and pituitary). The model is trained and tested on a dataset consisting of 3,394 images, and the average accuracy is 98.22%. Finally, the proposed model results are compared with well-known techniques such as VGG16, VGG19, and CNN-SVM.

Rizwan et al. [37] proposed Gaussian Convolutional Neural Network (GCNN) for brain tumor classification and used many pre-processing filters to enhance the classification task. The proposed method is trained and tested on two datasets. The first dataset is brain tumor dataset [38] and the second dataset from “The Cancer Imaging Archive (TCIA)" [39]. The proposed method achieved an accuracy of 99.8% and 97.14% on both datasets respectively.

Chattopadhyay and Maitra [40] proposed a CNN model for detecting brain tumors from magnetic resonance imaging. The model consists of two Convolution layers with activation function Relu. Each Convolution layer follows a batch normalization layer and a maxpooling layer. The model has two fully connected layers, the first layer has the ReLU activation function whereas the second one has a Softmax activation function. The model is trained and tested on the BraTS 2020 dataset, and achieved 99.74% accuracy.

Jun and Liyuan [41] developed a new model for classifying brain tumors using magnetic resonance imaging. The proposed model integrates an attention mechanism focusing on important information with a multipath network to improve accuracy. The model achieved an accuracy of 98.61% when it was tested using a brain tumor dataset [38].

Yin et al. [42] introduced CEFormer, a Convolution-based Efficient Transformer that integrates depthwise and dilated convolutions with attention to improve feature extraction. Their hybrid design achieved good accuracy and faster convergence, balancing performance and computational efficiency.

Hong et al. [43] suggested an upgraded Vision Transformer (VIT-B/16) model to enhance diagnostic precision. The work addresses the limits of the dataset by utilising image-enhancing techniques such as homomorphic filtering, channel contrast limited adaptive histogram equalization, and unsharp masking. These techniques enhance the dataset and enhance the generalisation of the model. The work presents a new technique for encoding relative positions to overcome the constraint of the Vision Transformer in capturing input token sequences and improving its prediction abilities. Incorporating residual structures in the Multi-Layer Perceptron (MLP) speeds up convergence and improves accuracy. The suggested model achieves a classification accuracy of 91.36% on an upgraded open-source brain tumour dataset, surpassing the original VIT-B/16 by 5.54%.

Gunasekaran et al. [44] proposed a hybrid deep learning model combining ConvNet with ResNeXt10 for automated brain tumor segmentation and classification using MRI. The most informative features are selected via Advanced Whale Optimization (AWO). The proposed model is evaluated on the BRATS 2020 dataset, and achieved an accuracy of 99.27% for tumor core detection.

Gundogan [45] proposed a novel hybrid deep learning model combining a custom CNN feature extractor with the XGBoost classifier. It also includes the Grad-CAM method for the model’s decision-making explainability. The model achieved 99.77% accuracy over four classes (glioma, meningioma, pituitary, no tumor).

Filvantorkaman et al. [46] introduced an ensemble model combining MobileNetV2 and DenseNet121 via a soft voting strategy. The model integrated Grad-CAM++ and a symbolic clinical rule overlay for explainability. Evaluated on the MRI dataset, the model achieved 91.7% accuracy and a Dice score of 0.88.

### Based on standard transfer learning models

This section explores how researchers have effectively adapted pre-existing deep-learning architectures to improve brain tumor classification and segmentation. By employing transfer learning, these studies enhance the capabilities of standard models, making them more suitable for specific medical imaging challenges. For instance, Masood et al. [47] proposed brain tumor segmentation and precise classification using custom Mask Region-based CNN (Mask-RCNN) with a dense-net-41 backbone architecture trained by transfer learning. Two benchmark datasets are used to evaluate Mask-RCNN and the results show that the Mask-RCNN can accurately detect tumor and precise tumor locations. The model achieved an accuracy of 98.34% for classification and 96.3% for segmentation, however, the model is not tested on a large dataset to analyse the model robustness. The model is tested on two datasets, the first dataset contained 3064 MRI images whereas the second one contained only 253 MRI images, which are not sufficient to demonstrate the robustness of the model.

Noreen et al. [48] suggested a model of multi-level features extraction for brain tumors identification. This study presented two various scenarios. In the first scenario, features from various DensNet blocks are extracted from a trained DensNet201 model and passed to classify brain tumors using a softmax classifier. In the second scenario, the pre-trained Inceptionv3 model is used to extract features from the different Inception modules, which are passed to a softmax to diagnose the brain tumor. Publicly available tumor dataset is used to evaluate both scenarios, the results of the accuracy-test is 99.34% for Inception-v3 and 99.51% for DensNet20. The model has not been tested on a large database to evaluate the model robustness.

Rajinikanth et al. [49] developed a deep learning architecture (DLA) for the purpose of automated detection of brain tumors using magnetic resonance imaging. To discover brain diseases, they (i) suggested implementing a pre-trained deep learning structure such as (ResNet101, AlexNet, ResNet50, VGG19 and VGG16 ) and classification using SoftMax. (ii) using a pre-trained deep learning architecture but a decision tree, SVM-linear, SVM-RBF and k nearest neighbour (KNN) based on in-depth features for classification; (iii) and custom VGG19 with sequentially embedded features and other handcrafted features to improve accuracy. The results proved that VGG19 achieved better results compared with (VGG16, ResNet101, AlexNet, and ResNet50), where the VGG19 accuracy is of 97%, 98% 99% for the modalities T1C, T2, and Flair respectively. The study is limited to tumor detection and doesn’t deal with the classification of tumor type.

Raza et al. [50] proposed DeepTumorNet for brain tumor classification. The DeepTumorNet is a hybrid deep learning model based on the GoogLeNet model after removing the last five layers and adding 15 new layers instead of eliminating ones. they used a brain tumor dataset [38] to train and test the proposed model. The results were 99.67%, 99.6%, 100%, and 99.66% for accuracy, precision, recall, and F1-score, respectively.

Asif et al. [51] suggested a methodology for categorising brain tumors by utilising MRI scans. The researchers utilised deep transfer learning using five well-known architectures: Xception, DenseNet201, DenseNet121, ResNet152V2, and InceptionResNetV2. To improve classification accuracy, the last layers of these designs were adjusted by incorporating a deep, dense block and a softmax output layer. The model underwent testing using datasets that are accessible to the public. The results demonstrated exceptional performance, achieving an accuracy of 99.67% for a three-class dataset consisting of glioma, meningioma, and pituitary tumors and 95.87% for a four-class dataset that includes healthy patients.

Asif et al. [52] introduced a new and innovative deep learning model called BMRI-NET to enhance the accuracy of brain tumors classification from MRI data. The BMRI-NET model uses a deep-stacked ensemble method that combines three already-trained convolutional neural networks (CNNs): DenseNet201, ResNet152V2, and InceptionResNetV2. This ensemble method leverages the strengths of these models by combining their predictions to achieve higher accuracy and generalization. The performance of BMRI-NET was assessed using the Figshare brain MRI dataset, and the model achieved an accuracy of 98.69%.

Wong et al. [53] developed a CNN-based classification system using a pretrained VGG16 model. The model was trained and evaluated on brain MRI dataset (17,136 images), achieving 99.24% accuracy across four classes (glioma, meningioma, pituitary, and normal). Their approach proves the effectiveness of transfer learning in brain tumor classification tasks.

### Limitations of existing studies

While the development and application of deep learning models in MRI-based brain tumor detection have significantly advanced, several limitations remain that must be addressed for further improvement.

First, many studies rely on small datasets, which can lead to overfitting and reduced model generalizability. In addition to developing novel models from scratch, several studies have adapted existing deep learning architectures, such as VGG16, which were initially trained on large non-medical image databases like ImageNet. These pre-trained models are designed to recognize thousands of generic, non-medical images, which vary significantly in terms of colors, textures, and object types. Transferring learning from such non-medical domains to medical applications is a common strategy to address the challenges associated with data scarcity in medical imaging. However, despite the advantages of reduced training times and lower data requirements, this method introduces specific challenges: (1) Computational Cost: The complexity and depth of models like VGG16, designed for broad applications, can lead to higher computational demands when adapted for medical imaging. (2) Domain Specificity: The knowledge these models gain from non-medical images might not be entirely applicable to medical images. The fundamental differences in image characteristics between these domains can hinder the model’s performance. Recent studies, such as the research conducted by Alzubaidi et al. [54], have critically analyzed the benefits and limitations of using pretrained models on medical images. They empirically demonstrated that while pretrained models on ImageNet provide a valuable foundational knowledge base, their performance in medical domains can be substantially limited. The study highlights that in-domain transfer learning (TL)—where models are further trained and fine-tuned on medical-specific datasets—significantly improves performance compared to using pretrained models directly. This is due to the nature of medical imaging, where factors such as disease markers and tissue characteristics are vastly different from the objects and scenes found in standard image datasets.

Furthermore, many current studies lack a mechanism for explaining decisions and how the model made the decision, where decision-making processes are opaque and difficult for clinicians to interpret. This lack of transparency is particularly problematic in medical settings where understanding the basis for diagnostic or treatment decisions is critical to trust.

These limitations underscore the need for accurate, robust, interpretable, and efficient models. Our research aims to bridge these gaps by introducing the PRCnet model, which utilises novel architectural improvements to enhance performance. We designed the PRCnet model by performing many experiments to choose the optimal filter size and kernel size for each convolutional layer. We analyzed that using different filter kernel sizes with parallel layers increased the model’s accuracy. Also, we used connections between layers to obtain features from different levels and address the problem of gradient vanishing. Furthermore, a dropout layer is used to help overcome the problem of overfitting.

To address the issues of interpreting data and how the model makes decisions, we used explainable AI techniques such as Grad-CAM and layer visualization, which provide visual explanations of the model’s decision-making process. These techniques help demystify the internal processes of the model, allowing clinicians to understand the features that influence the model’s predictions. Incorporating AI not only alleviates transparency issues, but also enhances the power and reliability of deep learning models in clinical applications.

## Proposed methodology

**Fig 2:**
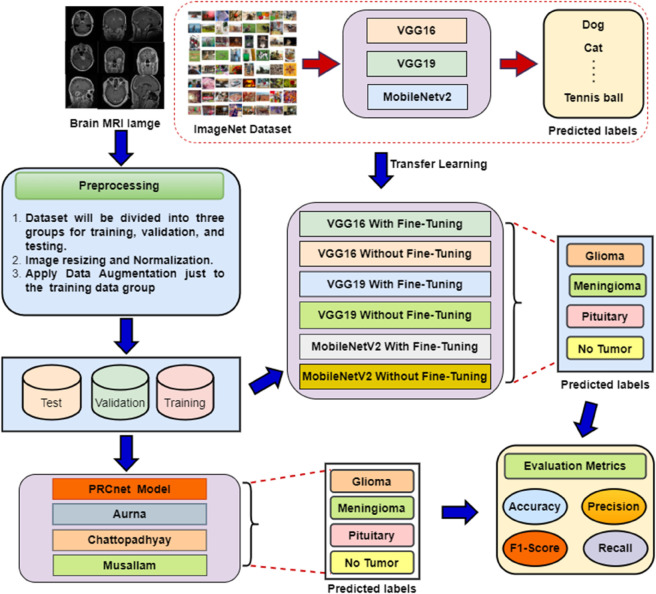
Flow of various steps including pre-processing, transfer learning, feature extraction, classification, and evaluation matrix.

This section includes the datasets used in this article and the preprocessing of the datasets and discusses the proposed PRCnet model. The Fig 2 shows a summary of the proposed methodology. In the first stage, the dataset of brain MRI images used for training and testing is determined namely dataset A and B. The second stage is data pre-processing (such as image resizing), afterwards, the images are divided into three training, validation, and testing sets. The third stage is to design the PRCnet model, and train / test it on datasets A and B. The fourth stage is to identify some of the most popular and pre-trained standard models (such as VGG16, VGG19 and MobileNetV2) and use the learning transfer from the ImageNet dataset, then train/test the models on dataset A and B. Moreover, we train and test some state-of-the-art relevant papers on dataset A and B. Finally, we compared the results of the PRCnet model with the results of the (VGG16, VGG19, MobileNetV2, Aurna [28], Chattopadhyay [40] and Musallam [30]) models.

### Datasets

**Fig 3:**
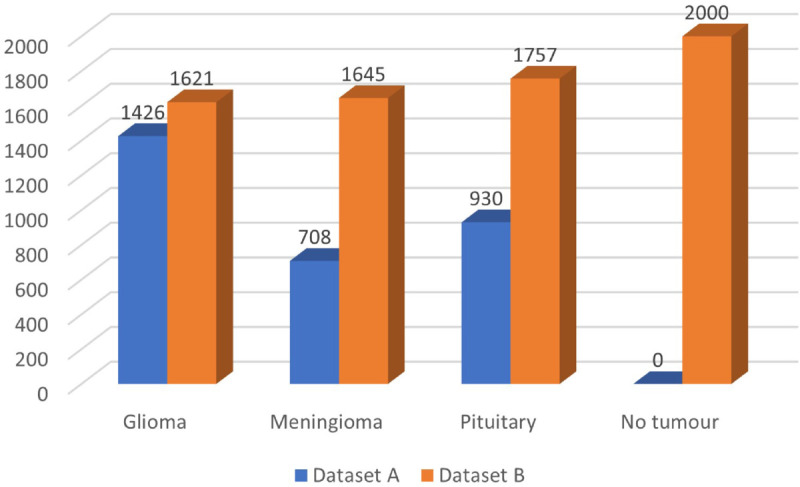
Distribution of brain tumor categories.

**Fig 4:**
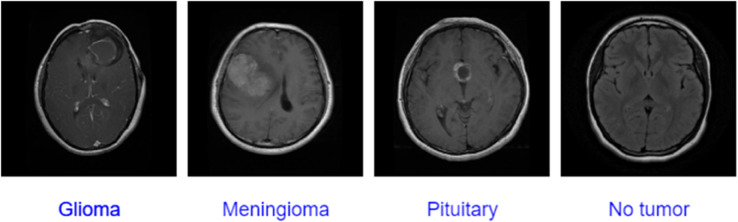
A sample from the dataset B that presented the types of tumors.

The first database (Dataset A) we used is the brain tumor dataset, introduced by Cheng et al. [38,55]. It has 3064 MRI images of the brain collected from 233 patients in different views, including sagittal, coronal, and axial. It is classified into three classes according to the types of brain tumors. It contains 708 images of meningioma, 930 images of pituitary tumor and 1426 images of glioma. Images are available in Mat format, and the size of the MRI images in this dataset is 512 x 512 pixels. The second dataset (Dataset B) is Brain Tumor MRI Dataset collected from the Kaggle website [56]. It contains 7023 labelled images with different sizes grouped into four categories: 1621 images of glioma tumors, 1645 images of meningioma tumors, 2000 images of no tumor, and 1757 images of a pituitary tumor. Fig 3 shows the distribution of brain tumor categories for both datasets. Moreover, Fig 4 presented a sample from the dataset that contains four categories of MRI.

### Pre-processing

All images in both data sets are resized to 224 x 224 to fit for the PRCnet, VGG16, VGG19 and mobileNetV2 input constraint. Then dataset A is divided into three groups 70% of the images are for training, 15% is validation, and the remaining 15% are for testing. On the other hand, dataset B is divided into three groups also 80% for training, 10% for the validation and 10% for the testing.

We used data augmentation techniques to increase the training dataset to overcome the lack of data for training. Many augmentation methods increase the training data set, which helps overcome the over-fitting problem. The augmentation techniques used in this paper are brightness, rotation, horizontal flip, vertical flip and shift.

### The PRCnet model

This paper proposes an efficient CNN architecture to detect and classify brain tumors in MRI images.The PRCnet model used different filter sizes with parallel convolution layers also residual connections. The PRCnet model architecture mainly consists of convolution layers, max pooling, batch normalization and a fully connected layer. We chose a hyperparameter for the PRCnet model through several experiments. The optimal one was chosen after a random search was conducted using Keras Tuner on different sets of hyperparameters. Then, the best hyperparameters results chosen by the random search were compared with what was selected manually. Table 1 shows the values of the hyperparameters and the values that were chosen. Table 2 shows hyperparameter values and results obtained for the best eight models out of 20 trials, and each trial was tested twice. Also, Table 2 shows the results obtained through manual hyperparameter values selection. The test was conducted on dataset B which is divided into two groups 80% for training and 20% for validation. The Batch size is set to 16, and the Epoch size is 30.

**Table 1:** Hyperparameters search space and which value is selected for PCRnet model.

Hyperparameter	Search space	Value chosen
No filters in Conv 1	32, 64 or 128	**32**
Kernel size of Conv 1	3,5 or 7	**3**
No filters in Conv 2	32, 64 or 128	**64**
Kernel size of Conv 2	3,5 or 7	**3**
No filters in Conv 3	32, 64 or 128	**64**
Kernel size of Conv 3	3,5 or 7	**3**
No filters in Conv 4	32, 64 or 128	**64**
Kernel size of Conv 4	3,5 or 7	**5**
No filters in Conv 5	32, 64 or 128	**64**
Kernel size of Conv 5	3,5 or 7	**7**
No filters in Conv 6	32, 64 or 128	**128**
Kernel size of Conv 6	3,5 or 7	**3**
No filters in Conv 7	32, 64 or 128	**128**
Kernel size of Conv 7	3,5 or 7	**3**
No filters in Conv 8	32, 64 or 128	**128**
Kernel size of Conv 8	3,5 or 7	**5**
No filters in Conv 9	32, 64 or 128	**128**
Kernel size of Conv 9	3,5 or 7	**7**
No filters in Conv 10	64, 128 or 256	**256**
Kernel size of Conv 10	3,5 or 7	**3**
No filters in Conv 11	64, 128 or 256	**256**
Kernel size of Conv 11	3,5 or 7	**3**
No filters in Conv 12	64, 128 or 256	**256**
Kernel size of Conv 12	3,5 or 7	**3**
No filters in Conv 13	64, 128 or 256	**256**
Kernel size of Conv 13	3,5 or 7	**3**
No filters in Conv 14	64, 128 or 256	**256**
Kernel size of Conv 14	3,5 or 7	**5**
No filters in Conv 15	64, 128 or 256	**256**
Kernel size of Conv 15	3,5 or 7	**7**
No. Units in Dense layer 1	min value:256,max value:1024	**256**
No. Units in Dense layer 2	min value:256,max value:1024	**256**
Learning rate	min value: 0.0001,max value:0.01	**0.0001**

**Table 2:** Hyperparameter values for the highest eight models accuracy results out of 20 trials as well as the results through manual hyperparameter values selection.

Hyperparameter	Trial 1	Trial 2	Trial 3	Trial 4	Trial 5	Trial 6	Trial 7	Trial 8	Manual selection
No filters in Conv 1	32	128	128	32	128	32	64	32	**32**
Kernel size of Conv 1	7	7	5	5	7	7	7	7	**3**
No filters in Conv 2	64	64	32	32	128	64	64	64	**64**
Kernel size of Conv 2	7	3	3	5	5	3	3	5	**3**
No filters in Conv 3	64	128	128	32	64	128	128	64	**64**
Kernel size of Conv 3	5	7	5	7	5	3	3	5	**3**
No filters in Conv 4	128	64	32	32	32	128	64	32	**64**
Kernel size of Conv 4	7	5	3	3	7	7	3	7	**5**
No filters in Conv 5	128	128	64	128	128	32	64	64	64
Kernel size of Conv 5	7	5	7	3	3	3	3	5	**7**
No filters in Conv 6	128	256	64	256	256	64	128	256	**128**
Kernel size of Conv 6	5	7	5	7	5	3	7	7	**3**
No filters in Conv 7	64	128	256	128	64	64	256	64	**128**
Kernel size of Conv 7	3	5	5	7	7	7	7	7	**3**
No filters in Conv 8	256	64	256	64	64	256	256	64	**128**
Kernel size of Conv 8	7	3	3	5	7	7	3	7	**5**
No filters in Conv 9	128	256	128	128	256	128	256	256	**128**
Kernel size of Conv 9	3	3	7	3	7	5	3	5	**7**
No filters in Conv 10	256	64	128	64	64	64	256	64	**256**
Kernel size of Conv 10	5	7	3	3	5	7	5	5	**3**
No filters in Conv 11	256	256	128	128	128	128	256	128	**256**
Kernel size of Conv 11	7	3	7	5	7	7	5	5	**3**
No filters in Conv 12	128	64	64	64	128	64	128	64	**256**
Kernel size of Conv 12	5	7	3	7	7	5	7	5	**3**
No filters in Conv 13	64	64	64	256	256	64	256	64	**256**
Kernel size of Conv 13	3	7	7	3	7	3	7	7	**3**
No filters in Conv 14	256	256	256	64	128	256	128	64	**256**
Kernel size of Conv 14	7	7	5	7	7	5	7	5	**5**
No filters in Conv 15	64	256	256	64	64	64	256	256	**256**
Kernel size of Conv 15	5	3	3	3	3	7	3	7	**7**
No. Units in Dense layer 1	768	1024	256	1024	768	384	1024	640	**256**
No. Units in Dense layer 2	640	384	256	384	896	256	640	512	**256**
Learning rate	0.001547	0.000535	0.002151	0.001764	0.001934	0.001197	0.000120	0.001566	**0.0001**
Validation Accuracy	0.8935	0.8787	0.8752	0.8718	0.8680	0.8618	0.8577	0.8390	**0.9100**

**Fig 5:**
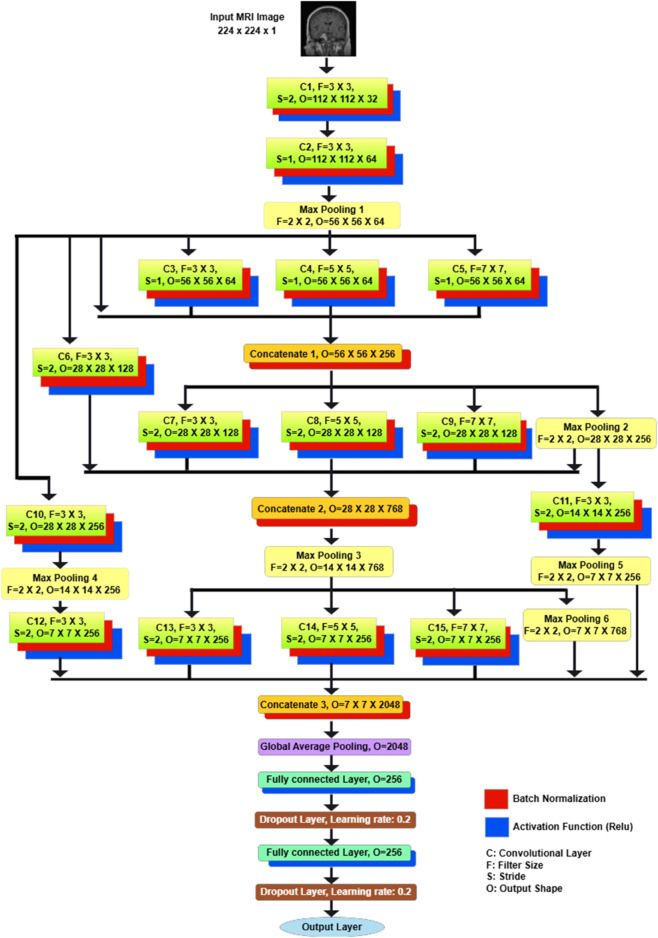
The architecture of PRCnet model.

See Fig 5 where the model is explained in detail. The size of the entered image is *224 x 224 x 1*. Starts a model with a convolutional layer with a filter size of *3 x 3)* and stride size of *2*. If we assume that the input image is I and the filter is K and its size *n x m*. The output of the convolution will be given by the equation [57].

$$s\left(i,j\right)=\sum_{m}\sum_{n}I\left(i-m,j-n\right)K\left(m,n\right)$$ (1)

Where s is the output image, I is the input image, k is the kernel filter and n,m is the size of the kernal filter.

Each convolutional layer is followed by a batch normalization layer for speeding up the training process. Whereas, the batch normalization layer normalizes the input for each small batch and propagates the gradients through normalization parameters, as shown in equation [58].

$$zi=y\frac{xi-m}{s}+b$$ (2)

Here *xi* is the input value, *y* and b learned parameter, *m* is the batch mean, s is the batch standard deviation. The Relu activation function will be applied after batch normalization to reduce the effect of the vanishing gradient problem. The first convolutional layer is followed by a second convolutional layer with a filter size of *3 x 3* and a stride size of *2*. The second layer follows a Max Pooling layer with a filter size of *2 x 2*. Then comes three blocks of parallel convolutional layers. Each block consists of three parallel convolutional layers with filter sizes *(3 x 3, 5 x 5, 7 x 7)* the use of different filter sizes to ensure that the model recognizes small and large features. The stride size in the first block is one, and in the second and third block is *2*. These three layers are combined with the connections from the previous layers with the concatenation layer followed by batch normalization and the Max Pooling layer after the second block. There are six connections between layers, some of which are short and direct, and some of the connections go through convolutional layers or Max Pooling or both. These connections are for a better representation of features, obtaining features from different levels, and addressing the problem of gradient vanishing. After that, global average pooling will be applied to reduce the input dimensions size to one dimension. Finally, there are three fully connected layers, the first and second layers with the ReLU activation function, and the last layer is for the classification with a Softmax activation function. After the first and second fully connected layers, a dropout layer with a ratio of 0.2. Dropout layer help to avoid the over fitting problem.

## Experiment results

In this section, we will present the experimental setup, adopted hyperparameters, evaluation metrics, and the results of testing the PRCnet model on the two datasets and comparing our results with other models.

### Experimental setup

To implement the proposed model, we used the python 3.8.0 programming language, Keras 2.6.0, and Tensorflow 2.6.0 libraries. Specifications of the computer that was used is: Intel(R) Core(TM) i7-10750H CPU @ 2.60GHz 2.59 GHz, 16GB RAM, NVIDIA GeForce GTX 1660 Ti GPU and Windows 10 installed.

### Hyperparameters

Hyperparameters are the basic things that must be determined before starting the training process, including Batch size, Epoch size, and the learning rate, where the Batch size is set to 64, Epoch size is 250, and the learning rate is 0.0001. Moreover, the early stop was set to stop the training if there is no improvement in the validation Loss after 40 Epoch and store the weights that achieved minimum validation Loss.

### Evaluation metrics

Performance measures play an essential role in testing and evaluation in developing any machine learning model [59]. Furthermore, evaluation metrics are essential for measuring the accuracy of any classifier, as the results of any classifier may be good against specific metrics and may not be good or bad against other metrics [60]. Therefore, metrics should be used in the training and testing phases to evaluate the system. Here are some common metrics used to evaluate the system [61,62].

1. **Accuracy**: The ratio of correct predictions is divided by the number of predictions evaluated.
  $$Accuracy=\frac{\left(TP+TN\right)}{\left(TP+TN+FP+FN\right)}$$ (3)
2. **Recall**: It is used to measure positive patterns that have been correctly categorized as in the following equation.
  $$Recall=\frac{TP}{\left(TP+FN\right)}$$ (4)
3. **Precision**: It is used to measure the proportion of positive patterns correctly predicted from the sum of the expected patterns in the positive category only.
  $$Precision=\frac{TP}{\left(TP+FP\right)}$$ (5)
4. **F1-Score**: It represents the harmonic mean between the recall and accuracy values.
  $$F1Score=2*\frac{\left(Precision*Recall\right)}{\left(Precision+Recall\right)}$$ (6)

where,*TN*= True Negative, *FN* = False Negative, *TP* = True Positive, and *FP* = False Positive.

### Results

In this experiment, we evaluated the PRCnet model using two datasets, Dataset A and Dataset B. Table 3 summarizes the testing results for both datasets, demonstrating the model’s robust performance with an accuracy of 94.77% on Dataset A and 97.1% on Dataset B. These results underscore the model’s capability to accurately classify brain tumors from MRI images. The effectiveness of the PRCnet model can be attributed to its use of parallel layers with filters of different sizes, which enable the capture of both small and large features. Additionally, the connections between different layers enhanced feature representation from various levels and address the issue of gradient vanishing, contributing to the model’s high performance.

**Table 3:** Results on the dataset A.

Dataset	Accuracy (%)	Precision (%)	Recall (%)	F1-Score (%)
Dataset A	94.77	94.73	94.77	94.74
Dataset B	97.10	97.18	97.10	97.09

**Fig 6:**
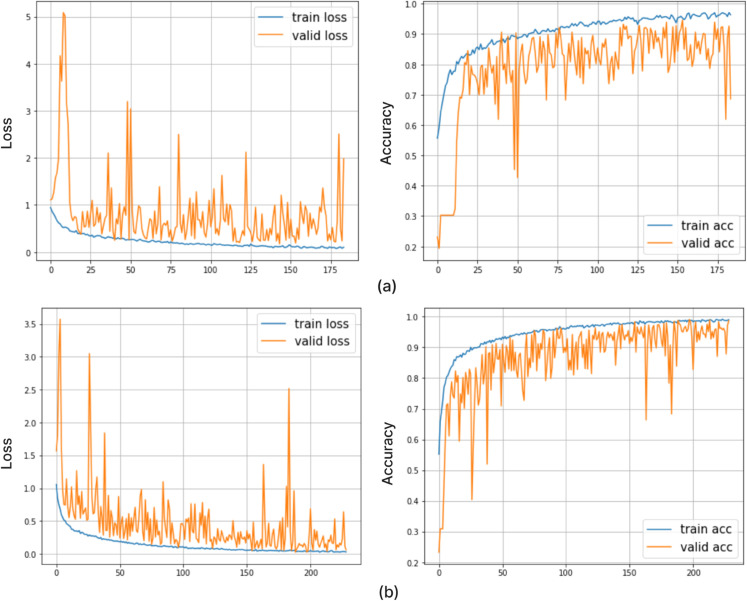
Loss and accuracy curves for the PRCnet model during training and validation for (a) dataset A and (b) dataset B.

Fig 6 shows the training and validation loss and accuracy curves over the training for both datasets. For dataset A (Fig 6 (a)): The loss curves show that the training loss decreases smoothly, indicating effective learning. However, the validation loss shows some fluctuations, which may indicate the model’s sensitivity to some data variance or slight overfitting. The accuracy curves reveal that the training and validation accuracy improves steadily as training progresses. For dataset B (Fig 6(b)): The loss for both training and validation decreases more consistently compared to dataset A, likely due to Dataset B’s larger size. The validation loss is relatively smoother, indicating more stable learning and better generalisation.

**Fig 7:**
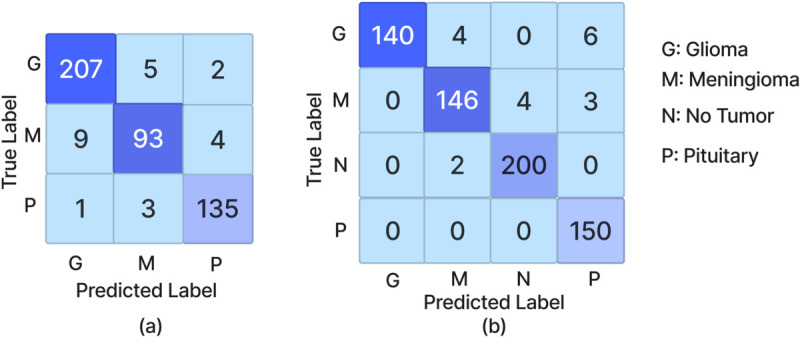
Confusion Matrices for the PRCnet Model on: (a) datasets A and (b) datasets B.

Fig 7 displays the confusion matrices, illustrating the classification performance of the PRCnet model across two datasets. For Dataset A (Fig 7 (a)), the matrix reveals high true positive counts for gliomas (207), meningiomas (93), and pituitary tumors (135), with minimal misclassifications, reflecting the model’s precision in detecting these tumor types. In Dataset B (Fig 7 (b)), the matrix shows high true positive counts for meningiomas (146) and pituitary tumors (150), along with high accuracy in classifying gliomas (140) and no tumor (200). The low numbers of false positives and false negatives across these matrices underscores the PRCnet model’s capability to effectively differentiate between various brain tumor types.

### Stratified K-fold cross validation

The cross-validation technique was used to verify the actual performance of the model and prove its robustness. The stratified K-Fold cross-validation method was used to validate the model. Where the data set is divided into k equal parts, the number of these parts depends on the size of the dataset [63,64]. In this study, we determined the value of k = 5. After the data set has been divided into five parts, each part is considered a test set and the rest are considered training. Table 4 shows the results of testing the PRCnet model performance on datasets A and B using the stratified K-Fold cross-validation technique.

**Table 4:** Performance of the PRCnet model after applying stratified K-Fold cross-validation.

Dataset	Fold	Accuracy (%)	Precision (%)	Recall (%)	F1-Score (%)
	Fold 1	93.64	93.76	93.64	93.68
	Fold 2	95.76	96.09	95.76	95.82
Dataset A	Fold 3	94.45	94.68	94.45	94.52
	Fold 4	94.94	94.92	94.94	94.93
	Fold 5	91.18	91.47	91.18	91.27
	Fold 1	95.73	95.80	95.73	95.67
	Fold 2	96.30	96.31	96.30	96.29
Dataset B	Fold 3	96.65	96.71	96.65	96.66
	Fold 4	97.72	97.72	97.72	97.72
	Fold 5	96.65	96.71	96.65	96.60

### The effect of data augmentation on the results

Developing a robust and reliable model for the detection of brain tumors requires a large and diverse data set in order to train it. Therefore, the application of deep learning in the field of medical images is rather difficult due to the lack of labeled training data [65]. In order to reduce the impact of lack of data in the proposed model, data augmentation was used, as five methods were used to increase the data as we mentioned in the pre-processing stage. The methods of augmentation were applied to the training data. The model was tested without the methods of augmentation and with the methods of augmentation, where the accuracy result was better with use the of data augmentation. Table 5 shows the accuracy of the model on both databases before and after using the data augmentation methods.

**Table 5:** The result before and after data augmentation.

Dataset	Accuracy (%) Before data augmentation	Accuracy (%) After data augmentation
Dataset B	93.74	**97.1**
Dataset A	93.46	**94.77**

### The effect of convolution filter size on the results

In order to test the effect of filter size on the accuracy of the model, the model was tested on different filter sizes, as shown in the Table 6. Experiments proved that the use of filters of different sizes gave better results because the use of different filter sizes helps the model to practice extracting large and small features.

**Table 6:** The result with a different filter size.

Dataset	filter size	Accuracy (%)
Dataset B	3 x 3	92.37
	5 x 5	96.49
	7 x 7	96.49
	Multi filter size (3 x 3, 5 x 5, 7 x 7)	**97.1**
Dataset A	3 x 3	91.5
	5 x 5	91.72
	7 x 7	93.46
	Multi filter size (3 x 3, 5 x 5, 7 x 7)	**94.77**

### Computational efficiency

To evaluate the computational cost of the PRCnet model, we monitored its training time and GPU memory usage. On average, the model required less than one minute per epoch (batch size of 64, 90 steps per epoch), with the full training completed within a few hours. The peak GPU memory consumption during training remained below 4 GB, confirming the computational efficiency of PRCnet.

### Explainability

The inherent “black box" nature of AI algorithms often raises concerns, particularly in medicine, where decisions have significant implications for patient care. Clinicians are hesitant to rely on AI-generated outcomes without a clear understanding of the decision-making process behind these outcomes [66]. Transparency and explainability are therefore paramount in deploying AI technologies in healthcare settings, as they provide clinicians with the necessary insights to evaluate and trust AI’s diagnostic processes.

**Fig 8:**
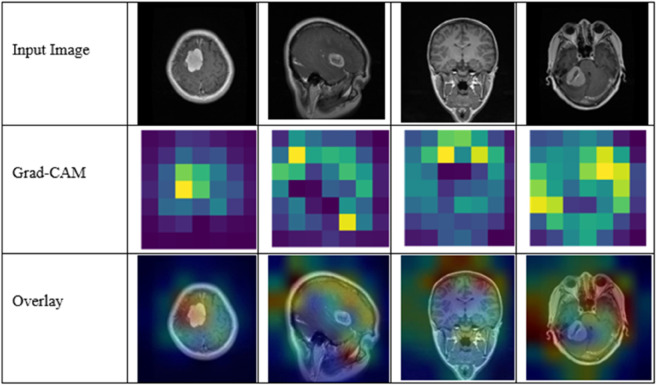
Grad-CAM visualization of features using PRCnet model.

To enhance the explainability of the PRCnet model, we incorporated Grad-CAM (Gradient-weighted Class Activation Mapping) visualization technology [67]. This technique was applied to the last convolutional layer of the PRCnet model to generate heat maps that visually depict the regions of the image most influential in the model’s predictions. As shown in Fig 8, the model consistently focuses on tumor-relevant areas rather than background structures, thereby supporting transparency and interpretability in clinical use.

**Fig 9:**
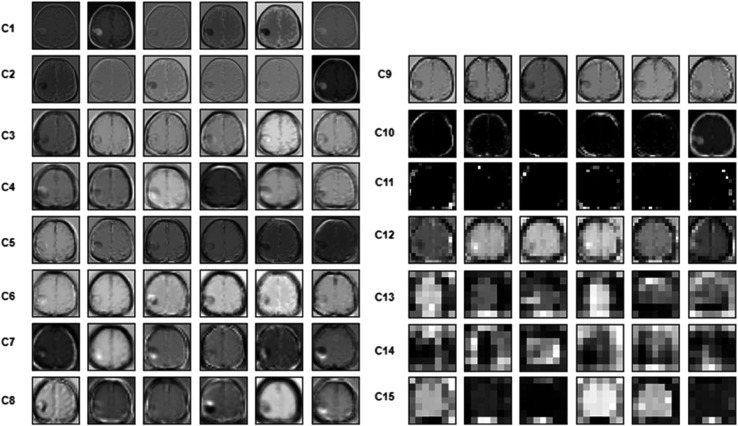
Samples visualization of six filters from each convolutional layer, C: convolutional layer.

Additionally, Fig 9 presents the impact of different convolutional layers on the processed images, with visualizations of six representative filters from each layer displayed. This visualization helps elucidate how each layer contributes to feature extraction, enhancing our understanding of the model’s decision-making process.

### Comparison of the PRCnet model with other models

In this study, we conducted a comprehensive comparison of the PRCnet model with the other models, including (VGG16 [31], VGG19 [31], MobileNetV2 [68], Aurna et al [28], Chattopadhyay et al [40], Musallam et al [30], TumorDetNet [32]). The results demonstrate that the PRCnet model achieved an accuracy of 94.77% on Dataset A and 97.1% on Dataset B. Detailed results for Dataset A and Dataset B are presented in Tables 7 and 8, respectively.

**Table 7:** Results on the dataset A.

Models		Accuracy (%)	Precision (%)	Recall (%)	F1-Score (%)
VGG16	Without Fine-Tuning	87.8	87.8	87.8	87.77
	With Fine-Tuning	92.16	92.21	92.16	92.1
VGG19	Without Fine-Tuning	88.24	88.2	88.24	88.2
	With Fine-Tuning	88.24	89.19	88.24	88.52
MobileNetV2	Without Fine-Tuning	90.2	90.01	90.2	90.08
	With Fine-Tuning	90.85	90.99	90.85	90.91
Aurna et al [28]		92.59	92.54	92.59	92.47
Chattopadhyay et al [40]		83.44	83.18	83.44	83.17
Musallam et al [30]		80.39	79.66	80.39	79.94
Ullah et al. [32] (TumorDetNet model)		89.98	90.16	89.98	90.05
**The PRCnet Model**		**94.77**	**94.73**	**94.77**	**94.74**

**Table 8:** Results on the dataset B.

Models		Accuracy (%)	Precision (%)	Recall (%)	F1-Score (%)
VGG16	Without Fine-Tuning	84.89	86.58	84.89	84.28
	With Fine-Tuning	90.69	91.34	90.69	90.71
VGG19	Without Fine-Tuning	83.82	85.13	83.82	83.39
	With Fine-Tuning	90.08	90.31	90.08	89.98
MobileNetV2	Without Fine-Tuning	92.06	92.33	92.6	92.02
	With Fine-Tuning	93.59	93.89	93.59	93.55
Aurna et al [28]		94.2	94.57	94.2	94.23
Chattopadhyay et al [40]		76.95	77.12	76.95	76.44
Musallam et al [30]		85.8	87.48	85.8	85.4
Ullah et al. [32] (TumorDetNet model)		93.28	93.64	93.28	93.31
**The PRCnet Model**		**97.1**	**97.18**	**97.1**	**97.09**

We observed that standard models such as VGG16, VGG19, and MobileNetV2 showed improved performance with fine-tuning compared to their performance without it. This improvement is attributed to the deep nature of these models which require substantial amounts of data to train effectively; hence, fine-tuning helps them better adapt to the specific characteristics of medical imaging data.

Furthermore, while the transfer learning from the ImageNet dataset was employed to enhance the training of these standard models, the significant dissimilarity between natural images from ImageNet and medical MRI images often resulted in suboptimal performance. In contrast, the PRCnet model’s architecture is specifically tailored for medical imaging, utilizing parallel layers with filters of varying sizes to capture both small and big features effectively. This design allows for a more nuanced feature representation.

Additionally, other models like that of Chattopadhyay et al. [40], which contain only two convolutional layers, were less effective, particularly as the dataset size increased. Their simpler architectures are not sufficient for capturing the complex patterns in medical imaging data, resulting in lower accuracy. Conversely, the model proposed by Musallam et al. [30], is complex and the computational cost is high because the kernel size for all convolutional layers is 7, so the accuracy of this model on dataset A was less than the accuracy of dataset B.

Moreover, the PRCnet model’s use of interconnected layers aids in mitigating the problem of vanishing gradients—a common issue in training deeper networks. This aspect of the architecture not only improves learning efficiency but also ensures robust feature extraction across the network, contributing to the superior performance of the PRCnet model across all tested datasets.

**Fig 10:**
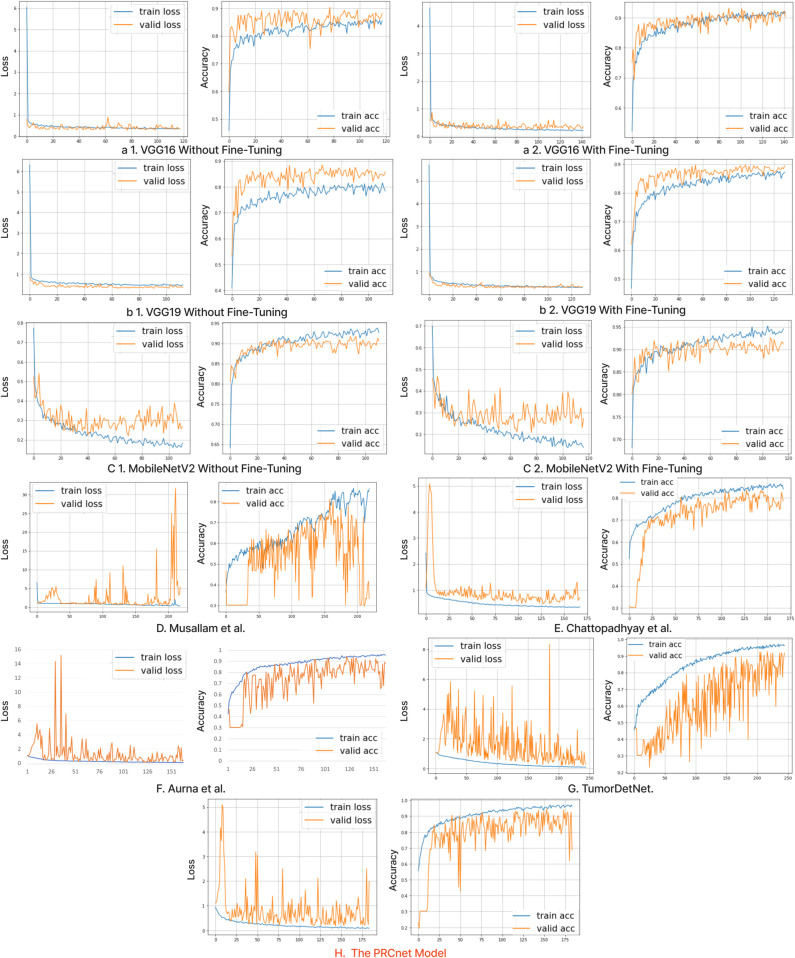
Losses and accuracy of all models during the training and validation process on the dataset A.

**Fig 11:**
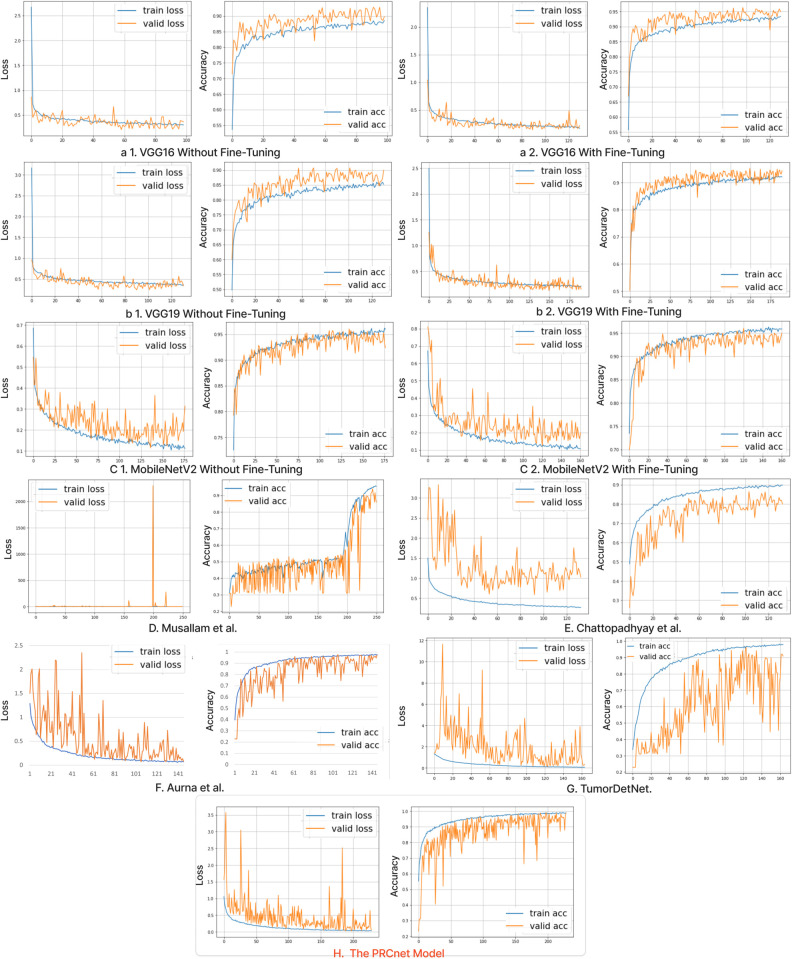
Losses and accuracy of all models during the training and validation process on the dataset B.

Fig 10 and 11 in the appendix illustrates the training and validation loss and accuracy curves for all models on Dataset A and Dataset B, respectively, emphasizing their learning patterns across epochs. While the designated number of epochs for all models is 250, the number may differ due to early stopping. This method terminates the training process when the validation performance no longer improves, preventing overfitting. Consequently, every model reaches its peak performance on the validation set at a distinct epoch, leading to separate stopping points for each model.

**Fig 12:**
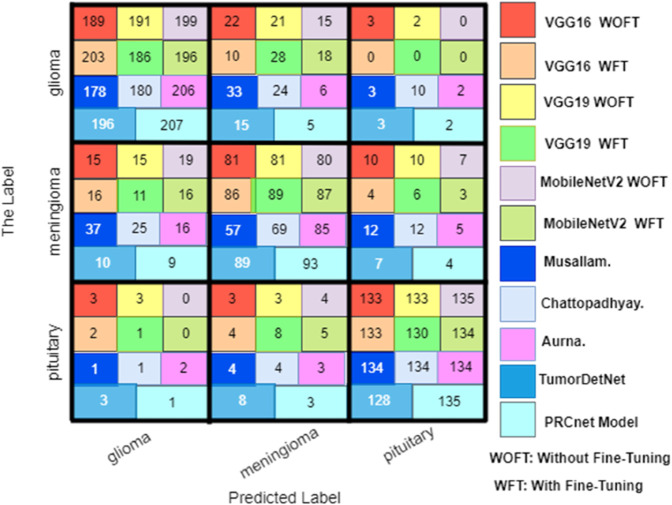
Confusion matrix for all models on the dataset A.

**Fig 13:**
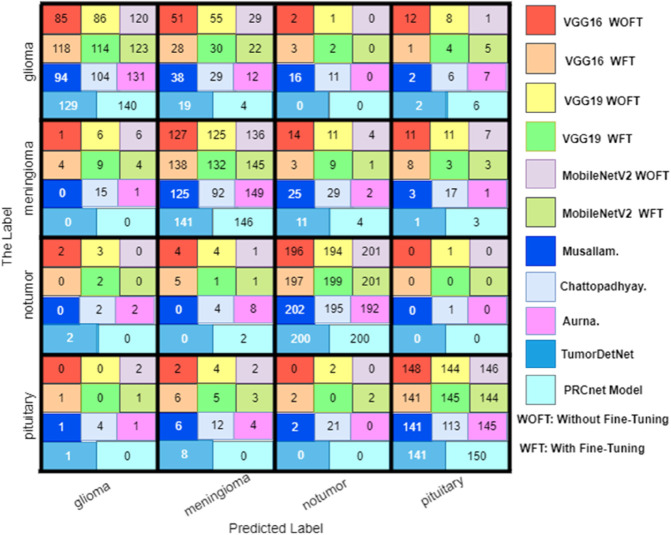
Confusion matrix for all models on the dataset B.

Fig 12 in the appendix provides a visual comparison via a confusion matrix of all tested models during the evaluation on Dataset A. While Fig 13 in the appendix displays the confusion matrix for all models during the testing process on Dataset B. These matrices allow for a direct observation of each model’s ability to correctly classify each tumor type, highlighting true positives and indicating any potential areas of misclassification.

## Discussion

In this study, we propose a PRCnet model for detecting brain tumors in MRI images. The PRCnet model uses parallel layers with different filter sizes to ensure that the model recognizes both small and large features in the MRI images. Additionally, the connection between different layers extracts features of different levels and helps overcome the gradient vanishing problem.

To evaluate the performance of the proposed PRCnet model, we conducted extensive experiments and comparative analyses on two datasets, namely Dataset A and Dataset B. The results of the experiments indicate that the proposed model achieved better results compared with the state-of-the-art models, with accuracy values of 94.77 and 97.1 for Dataset A and Dataset B, respectively.

We compared the results with the standard models, including VGG16, VGG19, and MobileNetV2, with and without fine-tuning. The accuracy of the standard models with fine-tuning on Dataset A is 92.16, 88.24, and 90.85 for VGG16, VGG19, and MobileNetV2, respectively. On the other hand, the accuracy of the standard models with fine-tuning on Dataset B is 90.69, 90.08, and 93.59 for VGG16, VGG19, and MobileNetV2, respectively. We used the transfer learning technique with standard models to obtain an initial weight from the ImageNet dataset and later fine-tuned it on the actual MRI image dataset. However, the proposed PRCnet model achieved better results than these standard models due to the deep of these models, which require a large dataset for training purposes. In addition, there is a significant difference between the ImageNet and MRI dataset content, negatively impacting the results.

We also compared the performance of the proposed model with the state-of-the-art models such as Aurna et al. [28], Chattopadhyay et al. [40], Musallam et al. [30] and TumorDetNet [32]. Where accuracy achieved by the models Aurna, Chattopadhyay, Musallam, TumorDetNet on Dataset A is 92.59, 83.44, 80.39 and 89.98, respectively. While accuracy achieved on Dataset B is 94.2 for Aurna, 76.95 for Chattopadhyay, 85.8 for Musallam, and 93.28 for TumorDetNet. The Musallam and Chattopadhyay models achieved lower accuracy than Aurna, TumorDetNet and PCRnet models. The reason why The Chattopadhyay and Musallam model has lower accuracy compared to others is the model complexity. The Chattopadhyay model contains only two convolutional layers, which is insufficient to extract all features, leading to lower accuracy. On the other hand, the Muslim model is computationally costly due to the kernel size of 7 for all convolutional layers and requires more training datasets, which is reflected in the accuracy results for both Dataset A and Dataset B.

The proposed PRCnet model has achieved a reasonable classification rate that can significantly impact clinical practice. By integrating PRCnet into existing diagnostic workflows, radiologists can achieve faster and more accurate detection of brain tumors, leading to timely and effective treatment plans. PRCnet can serve as a decision-support tool, providing a second opinion and increasing the confidence of medical professionals in their diagnoses. However, the model’s performance has not been validated in clinical practice, as the model’s performance may be affected by data variability from different sources. Therefore, future research should focus on training the model with more diverse datasets to further enhance its generalizability. Future research could also explore new data augmentation methods to improve model performance. Importantly, future studies should include more comprehensive clinical trials to evaluate the model’s performance in real-world scenarios. Additionally, predicting tumor location, grade, and size would be useful for analyzing medical images. This information could help physicians make more informed decisions about treatment options for patients.

## Conclusion

This paper proposes the PRCnet model for classifying brain tumors using magnetic resonance imaging (MRI). We utilized several techniques, such as parallel convolutional networks with different filter sizes, to achieve accurate and automatic classification to improve feature representation. In addition, connections between different layers enabled us to obtain features at different levels, overcoming the problem of gradient vanishing. We also employed global average pooling and dropout between the fully connected layer to mitigate overfitting issues. The PRCnet model was tested on two datasets, the results shows PRCnet significantly outperform the state-of-the-art models, achieving an accuracy of 97.1 on dataset B and 94.77 on dataset A respectively. These results demonstrate the efficiency and generalizability of the proposed model and its potential to aid in the accurate and speedy diagnosis of brain tumors. To further improve the performance and accuracy of the model, future work can focus on pre-training the model on larger datasets such as Imagenet and transfer learning. Additionally, data augmentation methods can aid in better training the model and addressing any data imbalance issues.

## Appendix

See Figs 10 to 13.
